# The “Bio-Crime Model” of Cross-Border Cooperation Among Veterinary Public Health, Justice, Law Enforcements, and Customs to Tackle the Illegal Animal Trade/Bio-Terrorism and to Prevent the Spread of Zoonotic Diseases Among Human Population

**DOI:** 10.3389/fvets.2020.593683

**Published:** 2020-11-03

**Authors:** Paolo Zucca, Marie-Christin Rossmann, Jorge E. Osorio, Kevin Karem, Paola De Benedictis, Josef Haißl, Paola De Franceschi, Elisa Calligaris, Michaela Kohlweiß, Giulio Meddi, Wolfgang Gabrutsch, Horst Mairitsch, Oronzo Greco, Roberto Furlani, Marcello Maggio, Massimiliano Tolomei, Alessandro Bremini, Ingrid Fischinger, Paolo Zambotto, Peter Wagner, Yvonne Millard, Manlio Palei, Gianna Zamaro

**Affiliations:** ^1^Central Directorate for Health, Social Policies, and Disabilities, Trieste, Italy; ^2^Bio-crime Veterinary Medical Intelligence Centre - c/o International Police and Custom Cooperation Centre, Thörl-Maglern, Austria; ^3^Agriculture, Forestry, Rural areas Veterinary Department, Land Carinthia, Klagenfurt, Austria; ^4^Department of Pathobiological Sciences, School of Veterinary Science, Madison, WI, United States; ^5^Centre for Global Health Leadership, Centre for Disease Control and Prevention, Atlanta, GA, United States; ^6^OIE Collaborating Centre and National Reference Centre for Infectious Diseases at the Animal-Human Interface, FAO and National Reference for Rabies, Istituto Zooprofilattico Sperimentale delle Venezie, Legnaro, Italy; ^7^Public Prosecutor Office, Klagenfurt, Austria; ^8^Public Prosecutor Office, Udine, Italy; ^9^Police Direction of the Land Carinthia, Klagenfurt, Austria; ^10^SCIP International Service of Police Cooperation, International Police and Custom Cooperation Centre, Thörl-Maglern, Austria; ^11^Custom Office, Ministry of Finance, Klagenfurt, Austria; ^12^Italian Financial Police, Regional Command Friuli Venezia Giulia Region, Trieste, Italy; ^13^Italian Army, Regional Command Friuli Venezia Giulia Region, Trieste, Italy; ^14^Smart Solution Mentor, Bologna, Italy; ^15^Veterinary Services, Autonomous Province of South Tyrol, Bolzano, Italy; ^16^Health and Care Management Department, Veterinary Services, Land Styria, Graz, Austria; ^17^Veterinary Services, Land Burgenland, Eisenstadt, Austria

**Keywords:** bio-crime, veterinary services, law enforcements, medical intelligence, zoonoses, illegal animal trade, bio-terrorism, cooperation

## Abstract

Illegal animal trade (pet, wildlife, animal products, etc.) is an example of transnational organized crime (T.O.C.) that generates a large business with huge profit margins. This criminal activity causes several negative effects on human health (zoonoses), animal health and welfare, market protection, consumer fraud and may be used as tool of agro/bio-terrorism. Illegal animal trade can facilitate the spread of zoonoses that are defined as diseases and infections that are transmitted by vertebrate animals to man. Humans are affected by more than 1,700 known pathogens: 60% of existing human infectious diseases are zoonotic and at least 75% of emerging infectious diseases of humans have an animal origin and 72% of zoonoses originate from wildlife or exotic animals. The Bio-Crime Project was developed in 2017 by Friuli Venezia Giulia Region (Italy) and Land Carinthia (Austria) together with other public institutions to combat illegal animal trade and to reduce the risk of disease transmission from animals to humans. Project partners agreed that a multi-agency approach was required to tackle the illegal animal trade that was high value, easy to undertake and transnational crime. The Bio-crime model of cross-border cooperation introduces the novel approach of replicating the cooperative framework given by the triad of Veterinary Public Health, Justice and Law Enforcements/Customs across borders using the International Police and Custom Cooperation Centres (IPCCCs) as a connection link among public entities of the neighbor countries. This model has been recognized as a best practice at European level because it can be easily replicated and scaled up without any supplementary cost for Member States.

## Introduction

The European Union (EU) faces an increasing level of transnational crime where criminal conduct in one country has an impact on another or even on many others. Illegal animal trade (pet, wildlife, animal products, etc.) is an example of transnational organized crime (T.O.C.) that generates a large business with great profit margins. In 2015, the cat and dog trade involved 61 million dogs and 67 million cats in twelve EU Member States, representing €1.3 billion and generated a direct employment of about 300,000 workers (1, 2). This criminal activity and its negative impacts in terms of risks to human and animal health, are considered by the European Commission to be a real and emerging risk for Member States and the need to improve legislation at the EU and national level on the welfare of dogs and cats involved in commercial activities has been underlined since 2013 (2, 3). Illegal pet sellers do not pay taxes or bear any costs necessary for ethical breeding/transportation of pets and the impact on the EU market of this criminal activity generates several negative effects on human health (zoonoses), animal health and welfare, market protection, consumer fraud and agro/bio-terrorism (1, 4).

### Zoonoses and Public Health

Illegal animal trade can facilitate a spread of zoonoses that are defined as diseases or infections that are transmitted by vertebrate animals to humans. In addition to being a public health problem, many of the major zoonoses prevent effective production of food of animal origin and create obstacles to the international trade in animal products (5). Humans are affected by more than 1,700 known pathogens: 60% of existing human infectious diseases are zoonotic. At least 75% of emerging infectious diseases of humans have an animal origin and 72% of zoonoses originate from wildlife or exotic animals. One factor leading to the emergence and spread of zoonotic infections is due to the increase of contacts between animals and humans that occurs for many disparate reasons (pets, food, population density, bush-meat, globalization and illegal animal trafficking) (6, 7). The trade in live animals and animal products is considered one of the major drivers of zoonotic disease emergence (8).

It is a mistake to think that the distribution and higher prevalence of zoonotic diseases is related to a higher density of mammalian hosts, meaning that the probability of contracting a zoonotic disease is higher in the tropical forests of the Old and New World. In fact, as reported by Han et al. (9) although major hotspots of mammalian hosts occur in the New and Old World tropics (South America and Eastern Africa, particularly), more zoonoses are concentrated in Northern latitudes, Eastern Africa, and Southeast Asia. According to these findings, it is very important to emphasize that zoonoses are not “exotic diseases” for North American and European citizens.

### Bio-Terrorism and Public Health

Bio-terrorism can be defined as the use of biological agents to intentionally produce disease or intoxication in susceptible populations - humans, animals, or plants - to meet terrorist aims. Biological agents may be naturally occurring or genetically modified and generally, the types of agents used as biological weapons cause systemic diseases, haemorrhagic fevers, pneumonias, or involve toxins and biological poisons (6, 10, 11). Biological weapons suitable for terrorist attacks are easy to produce, conceal, and transport. Elaborate “weaponization” is not needed for attacks to cause considerable damage and they have potential for dissemination over large geographic areas. Some pathogens can survive to sunlight, drying, heat, and they can cause high morbidity, mortality, resulting in public panic. Person-to-person transmission is possible for some infectious agents, many of which are difficult to diagnose and/or treat (6, 10, 11).

**Figure 1:**
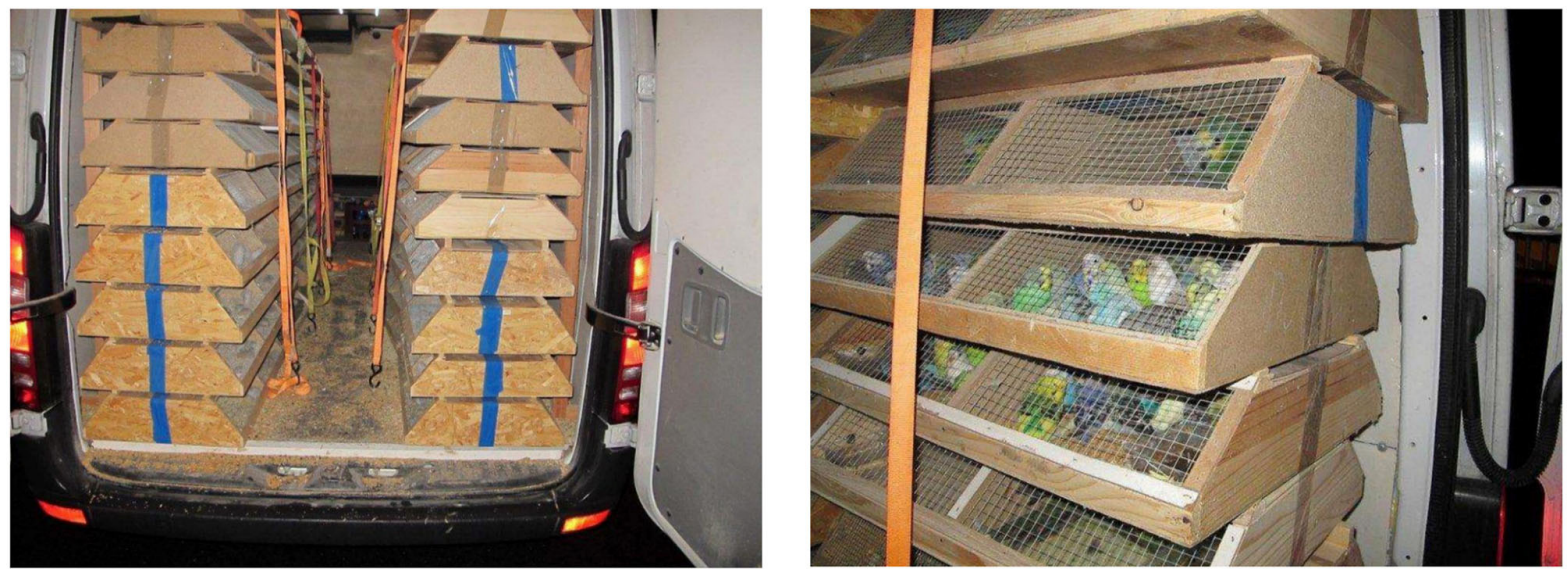
A truck with 2,615 parrots was confiscated on the Italian Austrian border of Tarvisio during a routine vehicle control by the Forestal Police of Friuli Venezia Giulia, December 2015. All the birds were infected with *Chlamydia psittaci* a zoonotic biosafety agent class III on IV of biosecurity level and recognized as a critical biological agent for public health. This agent can be transmissible by inhalation of contaminated dusts o contact with excretions but also by human-to-human infection (photos courtesy of Noava, Forestal Police of Friuli Venezia Giulia Region Italy).

Many naturally occurring pathogens could be used as well as genetically modified pathogens produced in a laboratory and this would make it difficult to discriminate between a naturally occurring outbreak and a bio-terrorist attack. In any case, regardless of whether the attack is related to a terroristic act or it is a naturally occurring outbreak, the damage caused by the biological agent is always significant for the affected country (see Figure 1).

### Legal Framework Concerning Zoonoses and Public Health

The EU system for monitoring and collecting information on zoonoses is based on the Zoonoses Directive 2003/99/EC (12), which obliges European Union Member States to collect relevant and, when applicable, comparable data on zoonoses, zoonotic agents, antimicrobial resistance and food-borne outbreaks (FBO) (13). Data collection on human diseases from Member States is conducted in accordance with Decision 1082/2013/EU (14) on serious cross-border threats to health. This Decision replaced Decision 2119/98/EC and identifies the fight against serious cross-border threats to health in response to planning and monitoring. Member States have to report data on infectious diseases to the European Centre for Disease Prevention and Control (ECDC) according to the Decision 2018/945/EU (15). Since 2008, data on human cases have been received by ECDC via The European Surveillance System.

The European Parliament Resolution 2019/2814 (16) of 12 February 2020 focuses on protecting the EU's internal market and consumer rights against the negative implications of illegal trade in companion animals. European Parliament Resolution P9 TA 0035 (17) approved on 14 February 2020 on a proposal from the Commission for the Environment, Public Health and Food Safety (SANI) indicates that profitable cooperation between Member States constitutes an urgent need and deems appropriate to promote and improve *ad hoc* training programs aimed at customs, medical and veterinary authorities in order to intercept pet illegal trade. This resolution underlines the importance of proposing training courses aimed at providing customs, medical and veterinary authorities with better tools for intercepting pet smuggling and highlights how Member States should ensure adequate staff training at borders.

### The Bio-Crime Project and the Bio-Crime Center

**Figure 2:**
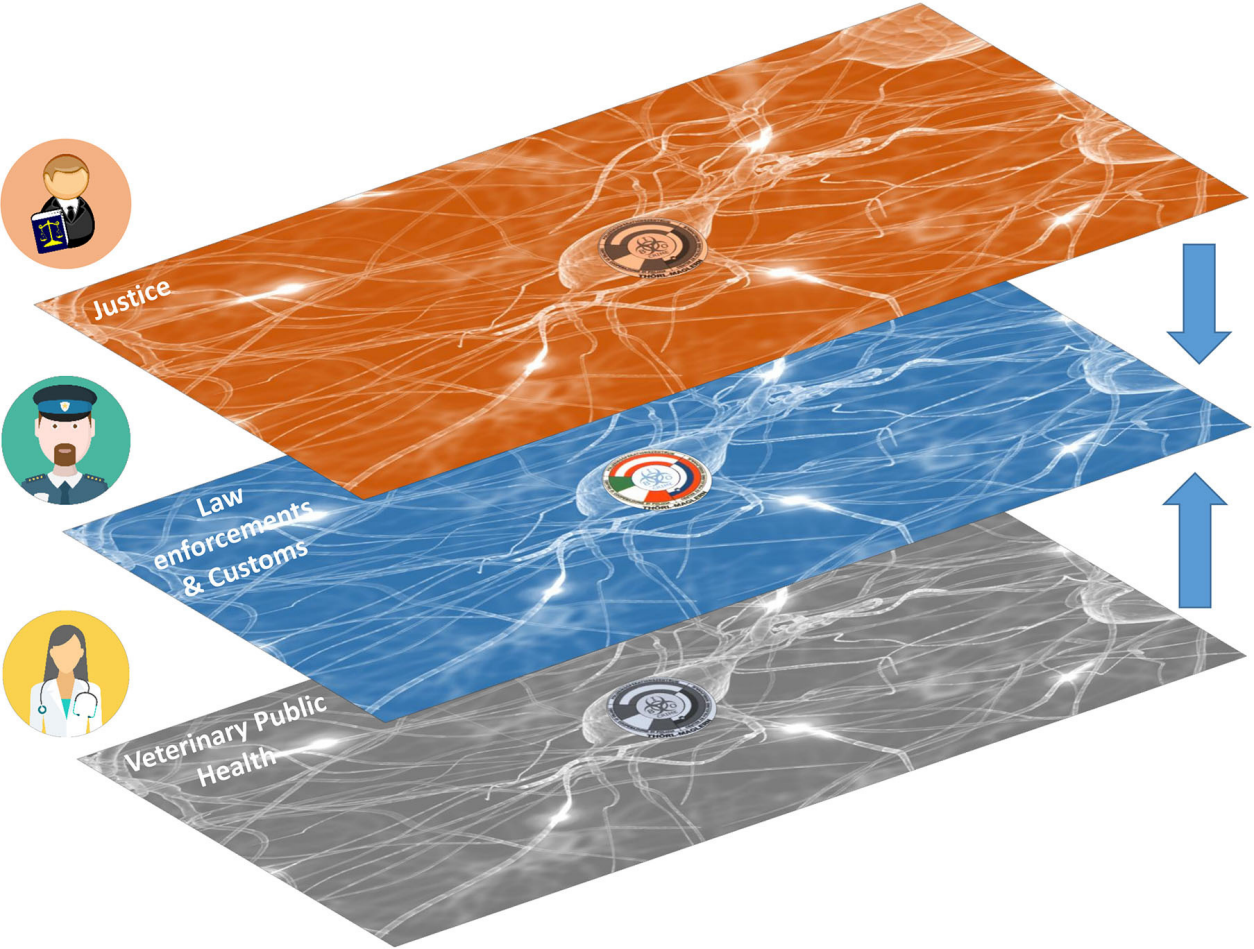
The International Police and Custom Cooperation Centre (IPCCC) of Thörl-Maglern (Austria) is the first IPCCC in Europe that host a connection link (synapse) among the Veterinary Public Health, Justice, Law Enforcements and Customs Networks (icons of Veterinarian, Policeman and Judge from Wikimedia Commons, the free media repository; synapse layer's patterns from Internet, source unknown, modified).

The two Regions on the border between Italy and Austria, Friuli Venezia Giulia and Carinthia, are both transit routes as regions of destination for the illegal animal trade from Eastern European countries (mainly pet animals such as dogs, cats, birds, small mammals, and reptiles). The Bio-Crime Project (18) (www.biocrime.org) was developed in 2017 by Friuli Venezia Giulia Region and Land Carinthia together with other public institutions to combat illegal animal trade and to reduce the risk of disease transmission from animals to humans, following numerous criminal episodes and zoonotic infections that had occurred in both Regions. The objective of this joint action funded by the European Community by means of the Interreg V-A financial tool, is to protect the health and safety of citizens through health prevention strategies and repression of cross-border crime. One of the project output that will maintains the sustainability of the actions after the end of the project is represented by the Bio-crime veterinary medical intelligence center that has been created by Friuli Venezia Giulia Region and Land Carinthia (19). This cross-border joint center put together all the public entities involved in tackling Transnational Organised Crime (TOC) related to the illegal animal trade and it is based into the International Police and Custom Cooperation Centre (IPCCC) of Thörl-Maglern (Austria). It is the first Veterinary Medical Intelligence Centre based inside an IPCC Centre in Europe. From a symbolic point of view, it represents the first neuronal synapse among the Veterinary Public Health, Justice and Law Enforcement/Customs Networks at a downside horizontal level (see also Figures 2, **4**).

### Public Entities Involved in Tackling TOC/Illegal Animal Trade and the Related Spread of Zoonoses

The public entities involved in tackling illegal animal trade and the related spread of zoonotic diseases among human population on the border between Italy and Austria, work within the framework of the Bio-crime project and belong to:

- Justice (Public Prosecutors Offices of Udine Italy and Klagenfurt Austria);
- Veterinary Services and Laboratories of Friuli Venezia Giulia Region Italy and Land Carinthia Austria;
- Law Enforcements and Customs of Italy and Austria;
- The International Police and Custom Cooperation Centre (IPCCC) of Thörl-Maglern.

An International Police and Custom Cooperation Centre (IPCCC) is an institution established by the EU national Police according to the Art. 39 of the Convention implementing the Schengen agreement for a better cross-border cooperation among the Law Enforcements and Customs of the Member States. In an International Police and Custom Cooperation Centre (IPCCC), officials from at least two, sometimes even several Member States, work together both in the office and in the field. On one hand, investigations by the security apparatuses of one State to another are processed directly by the respective officials in the cooperation center and allow efficient and rapid work, on the other hand a common field service is provided by patrolling the border area. The first International Police and Custom Cooperation Centre (IPCCC) among Austria, Italy and Slovenja was established in Thörl-Maglern in 2005 and it hosts the law enforcement representatives of Austria, Italy, Slovenja and Germany. At present there are more than 40 International Police and Custom Cooperation Centres (IPCCCs) distributed along the internal and external borders of the European Community.

### The Organizational Sector of Public Entities Involved in Tackling Transnational Organized Crime (TOC) and Illegal Animal Trade

**Figure 3:**
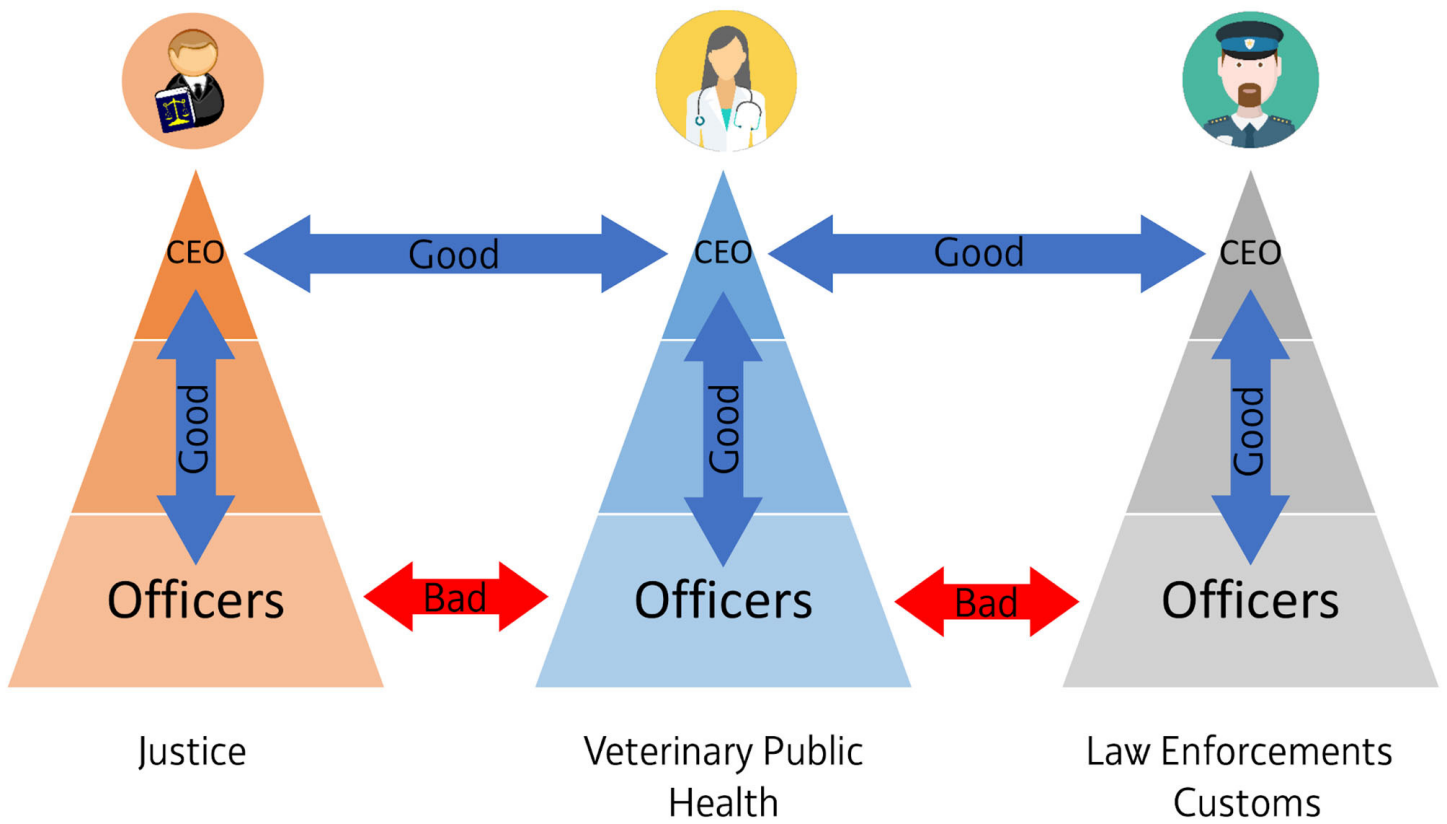
Organizational communication within and among public entities involved in tackling Transnational Organized Crime/Illegal animal trade. Vertical communication within a pyramidal structure is good although is time spending and slow down decision making (blue arrows). Horizontal communication among top management of different structures is good (blue arrows) while on the downside level, horizontal communication among Officers of different structures is poor (red arrows) (icons of Veterinarian, Policeman and Judge from Wikimedia Commons, the free media repository).

All the European public entities (Veterinary Public Health, Justice, Law Enforcements/Customs, IPCCCs) involved in tackling TOC/illegal animal trade are organized hierarchically top-down around vertical chains of command. This type of structure resembles a pyramid and gets wider as you move down. Roles are clearly defined within this structure, and every unit knows to whom it should report. However, on the downside, horizontal communication between different units may be poor, as the system is built around a vertical chain of command and furthermore all the different pyramid's layers of bureaucracy can slow down decision making and reduce the capability of the public entity to collaborate with other agencies and adapt to fast “environmental changes” (see Figure 3).

## Materials and Methods

Public entities belonging to the Veterinary Public Health, Justice, Law Enforcements/Customs and IPCCC of both side of the Italian-Austria border agreed that a multi-agency approach was required to tackle the illegal animal trade that was high value, easy to undertake and transnational crime. Such trade is endangering human and animal health by facilitating the spread of diseases and threatening economic security and public safety. In order to be effectively countered, this illegal trade requires a coordinated transnational response as well defined by "Decision no. 1082/2013 of the European Parliament and of the European Council on serious cross-border threats to health” that explain how preparedness and response planning are essential elements for effective monitoring, early warning and the fight against serious threats to cross-border health.

The Bio-crime cooperation model has been developed through a process of refinement of cross-border cooperation procedures between Friuli Venezia Giulia and Carinthia started in 2017. Initially, the increase in the exchange of information and cooperation between public entities at regional cross-border level occurred only between Veterinary Public Health services of Friuli Venezia Giulia Region (Italy) and Carinthia (Austria) that analyzed the problem and decided to enlarge the stakeholder's participation. Subsequently, the other two main stakeholders of the Bio-crime cooperative model represented by Justice and Law Enforcements/Customs were involved since only a rapid and horizontal communication that could take place also at the downside and horizontal level guaranteed an effective response to the contrast of the Transnational Organized Crime (TOC) and at the same time allowed the implementation of health prevention procedures for staff and citizens of the two Regions. The main connection link of the cross-border cooperation procedure was identified as the International Police and Custom Cooperation Centre (IPCCC) of Thörl-Maglern on the Italian-Austrian border that since December 2019 hosts the joint Friuli Venezia Giulia Region and Land Carinthia Bio-crime Veterinary Medical Intelligence Centre.

## Results

### Cross-Border Model of Cooperation

**Figure 4:**
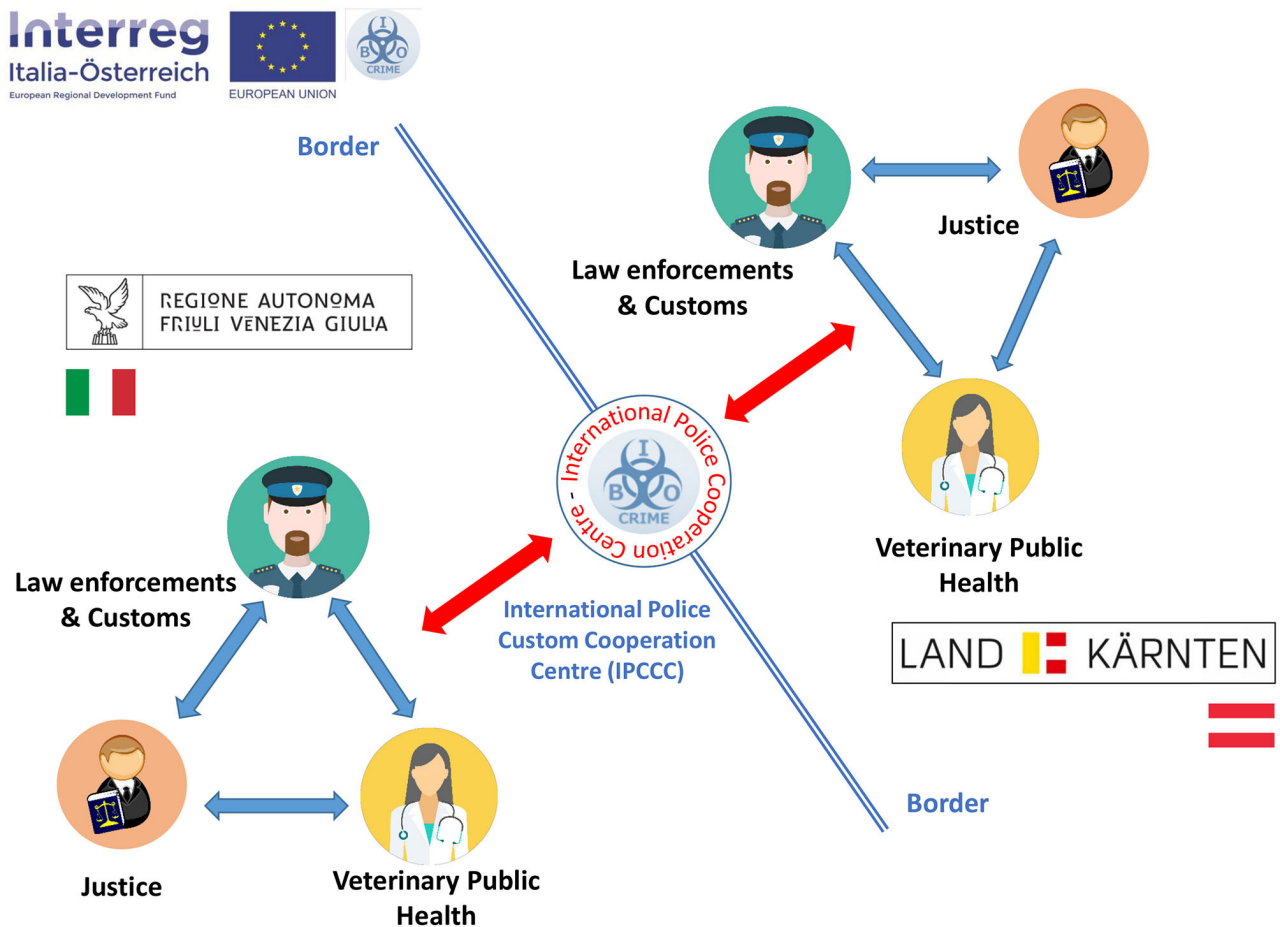
Bio-crime model of cross-border cooperation among Veterinary Public Health, Justice and Law Enforcements/Customs. The International Police and Custom Cooperation Centre (IPCCC) of Thörl-Maglern acts as the link between the two specular triad of public entities (icons of Veterinarian, Policeman and Judge from Wikimedia Commons, the free media repository).

The structural and functional architecture of the “Bio-crime model” of cooperation is described in Figure 4. One Region institutional triad represented by Veterinary Public Health, Justice, Law Enforcements/Customs is replicated specular on the other side of the border and the International Police and Custom Cooperation Centre (IPCCC) of Thörl-Maglern acts as the main connection link of the cooperation system. The IPCCC of Thörl-Maglern hosts the Bio-crime center office and two local branches at the Central Veterinary Directorates of Friuli Venezia Giulia and Carinthia in Trieste and Klagenfurt, respectively, have been established.

### Performance Indicators of Cross-Border Institutional Cooperation Before and After the Bio-Crime Program

The Bio-crime model has consolidated the institutional cooperation among five neighboring European Regions through the development of training programs for Law enforcements/Customs/Medical and Veterinary Officers, health prevention programs on biological risk/zoonoses in schools and development of Standard Operation Procedures (SOP) and best practices at a cross-border level. The different entities involved in the program exchange data directly during cross-border monthly meetings at the IPCCC of Thörl-Maglern or digitally via secure intranet institutional Share-Points. Furthermore, an alert system named “Bio-crime alert,” for short and fast communications within the partner's network has been established via an institutional mailing list. The performance indicators of institutional cross-border cooperation before and after the establishment of the Bio-crime program are summarized in Table 1.

**Table 1:** Performance indicators of cross-border institutional cooperation before and after the Bio-crime program.

**Public entities**	**Units**	**Before**	**After**	**Note**
Institutional cooperation established among Justice, Law Enforcements, Customs and Veterinary Services	Number	0	13	n. 2 Public Prosecutor Offices, n. 6 Law enforcements, Customs and Army, n. 5 Regional Veterinary Services
Regions involved	Number	0	5	Friuli Venezia Giulia, Land Carinthia, South Tyrol Province of Bolzano, Land Styria, Land Burgenland
**People**	**Units**	**Before**	**After**	**Note**
Law Enforcements/Public Officers trained during the past 3 years	Number	0	1.072	Customs and Law enforcements belonging to several different specialties, Medical and Veterinary Officers
Students involved into the health prevention program	Number	0	656^*^	Age cluster 11–13 years-old
**Standard Operation Procedures (SOP)**	**Units**	**Before**	**After**	**Targets**
SOP Public Prosecutors Offices (procedure for the correct execution of investigative activities/reports)	number	0	1	Law Enforcements/Customs, Veterinary Officers
SOP for Vehicle inspections (procedure for the correct and safe inspection of vehicles that might transport pet animals)	Number	0	1	Law Enforcements, Customs and Veterinary Officers
SOP Veterinary Public Health (procedure for the correct management of the confiscated animals /zoonotic risk)	Number	0	1	Public Prosecutors, Law Enforcements/Customs, Medical and Veterinary Officers

* *The school's program involved also students from Slovenja, Germany, Mauritius, and Japan*.

### Illegal Pet Trade Cross-Border Data Collection and Analysis Before and After the Bio-Crime Program

Prior to the development of the Bio-crime program, a comprehensive shared database on illegal pet trade data at cross-border level was not available. Over the years, the Bio-crime alert data sharing platform has made it possible to share information on the (i) country of origin, (ii) number of events, (iii) pet species prevalence, (iv) absolute numbers of dog and cat puppies confiscated, (v) prevalence of sanitary quarantine on the total number of pets, (vi) number of pets tested every year, (vii) diseases that are looked for through laboratory testing and a tendency indicator of the increase or decrease of the illegal pet trade over time (Table 2).

**Table 2:** Illegal pet trade data collection and analysis at cross-border level.

**Illegal pet trade data**	**Units**	**Before**	**After**	**Note**
Countries of origin	Number	Unkonwn^*^	13	Ukraine, Hungary, Romania, Slovakia, Poland, Bulgaria, Austria, Slovenja, Czech Republic, Serbia, Bosnia, Russia, China.
Events caught (Dec 2017–July 2020)	Number	Unkonwn^*^	40	Two peaks with four events per month in May 2018 and Sept 2019.
Pet species prevalence (Dec 2017–July 2020)	%	Unkonwn^*^	Dogs, cats, birds	In order of decreasing prevalence.
Puppy's dogs and cats confiscated (Dec 2017–July 2020)	Number	Unkonwn^*^	652	Maltese, French bulldog, Golden retriever, Terriers and Chihuahua puppies for dog breeds while Scottish folds for cats breeds show the higher prevalence among confiscated pets.
Sanitary quarantine on the total number of pets	Number	Unkonwn^*^	77.5%	Health documents or identification or country of origin data were missing.
Number of pets tested every year	Number	Unkonwn^*^	All	Until Dec. 2020.
Diseases that are looked for through laboratory testing	Category	Unkonwn^*^	Zoonotic and notifiable diseases	Among zoonotic diseases laboratories are focusing especially on Salmonellosis, Chlamydiosis, Rabies, endo-ectoparasites, and other notifiable diseases.
Illegal pet trade tendency indicator	Trend	Unkonwn^*^	Light increase	Very difficult to assess because at present many illegal pet transactions are placed via Internet and many pets are delivered directly from traders to buyers without any intermediate.

* *A comprehensive shared database at cross-border level was not available before the establishment of the Bio-crime program*.

**Figure 5:**
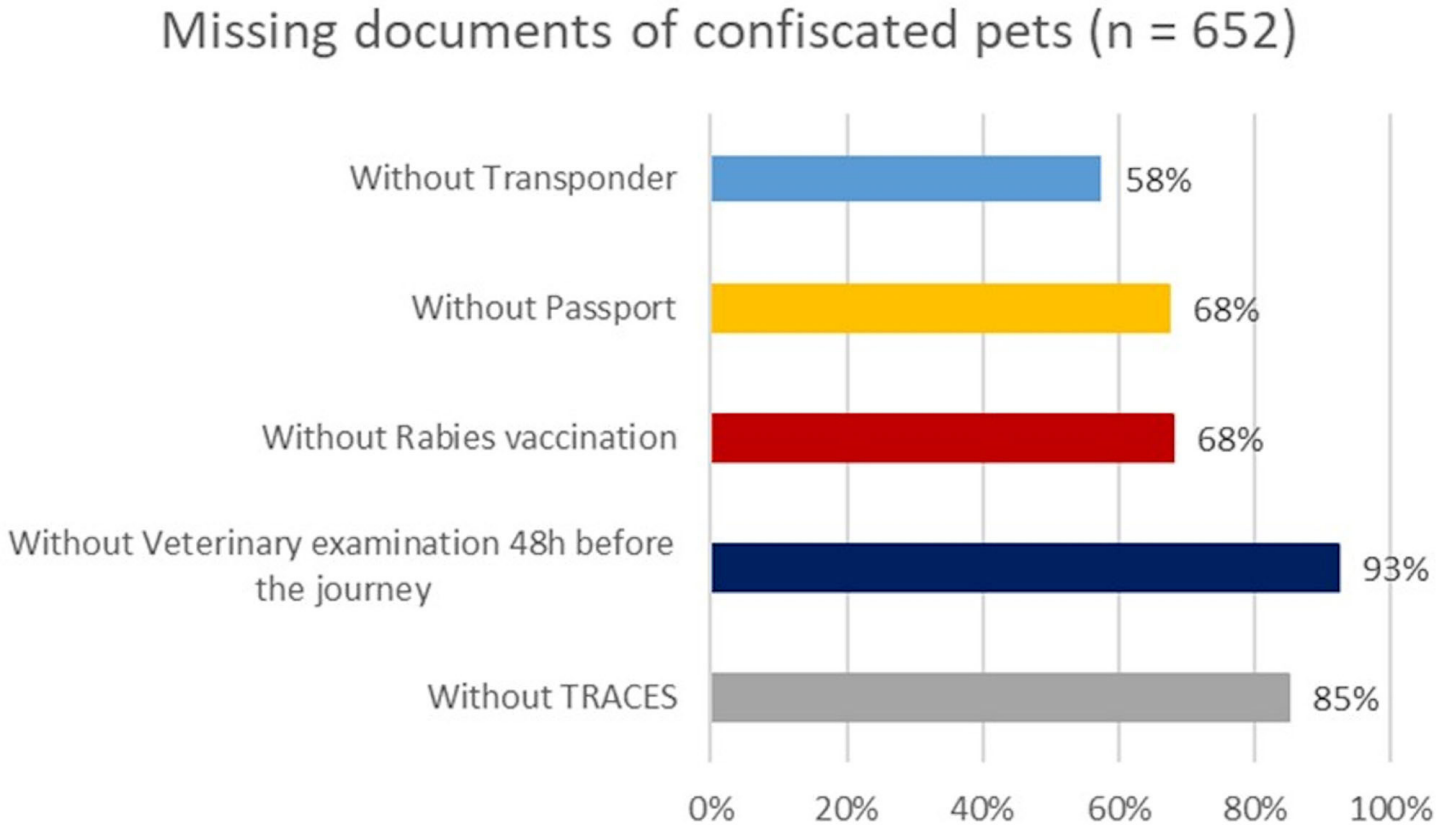
Missing documents of confiscated pets (dog and cat puppies *n* = 652) in Friuli Venezia Giulia Region Italy, Autonomous Province of South Tyrol Bolzano Italy and Land Carinthia Austria (Dec 2017–July 2020).

Furthermore, this dataset cross-checked with data reported on the documents that accompany the animals, with particular reference to the presence of a transponder for individual identification, the pet passport, the valid rabies vaccination certificate, of a clinical examination by a veterinarian within 48 h prior to departure and of the TRACES certificate, if relevant, as shown in Figure 5.

### The Cost-Benefit Ratio (CBR) of the Program Investments of the Bio-Crime Project

It is always complex to provide a quantitative analysis of the Cost-Benefit Ratio (CBR) applied to the areas of crime reduction and health prevention since the positive effects of preventive actions in these two areas are not easily quantifiable financially and they are often difficult to link to a specific geographical area. In order to provide the clearest picture of the CBR relating to the Bio-crime program, the analysis will focus on two specific project scenarios represented by (i) Free training courses on biological risks/zoonoses and by (ii) Border controls on illegal pet trade.

#### The Cost-Benefit Ratio (CBR) of Free Training Courses on Biological Risks/Zoonoses

A total number of 1,072 law enforcements, customs, medical and veterinary officers have attended the free training courses over the past 3 years. The average cost on the market of a 2-day training course on these subject amount to about €2,300. and therefore the cost avoidance for the public system for 1,072 units of personnel is €2,465,600. As the budget of the Bio-crime project Working Package (WP) 4 allotted to training courses was €120,000. the CBR amount to 19.57 meaning that the benefit was almost twenty times the budget cost.

#### The Cost-Benefit Ratio (CBR) of Border Controls on Illegal Pet Trade

The border controls for tackling illegal animal trade are not part of a specific Working Package (WP) of the project as they include personnel, administrative, travel costs, health screenings on confiscated animals, and training courses for staff members; to avoid any overestimation of the CBR, it was decided to consider the entire budget of the project (€1,117,300) as the overall cost of the project program. Taking as an example the scenario of 2,615 parrots infected with *Chlamydia psittaci* confiscated at the Italian-Austrian border, if these animals were sold on the market and assuming the best epidemiological scenario a low transmission rate between parrots and humans with absence of human-to-human transmission of the infection, the costs of the outbreak for the public health system would approximately be €35 M. The worst epidemiological scenario includes in the model the possibility of a human-to-human transmission of the pathogen with a basic reproductive rate *R*_0_ = 10 (20). The estimated costs of the outbreak for the health system could therefore rise up to €350 M (21). Assuming the best epidemiological scenario, the CBR would be 31.32 while for the worst it could reach 313.20.

## Discussion

### Cross-Border Model of Cooperation

There are several examples of joint cooperation programs among veterinary services and law enforcement/customs agencies all over the world although these programs don't normally involve a third important actor, i.e., the judiciary system. The Bio-crime model includes in its cooperative framework also public prosecutors whose contribution is essential to tackle the illegal animal trade and reduce the related health risks for citizens. Furthermore, this model introduces the novel approach of replicating the cooperative framework across borders using the International Police and Custom Cooperation Centres (IPCCCs) as a connection link among public entities of neighboring countries.

According to this new approach, at European level the “Bio-crime model” has been recognized as a best practice that can be easily replicated and scaled up without any supplementary cost for the Member States. In fact, the Veterinary Public Health, Justice, Law Enforcements/Customs and IPCCCs infrastructures already exist all over Europe. It is only a matter of involving all the public stakeholders and increasing the downside horizontal communication among different public entities of the framework. The model has been discussed during the conference “*Illegal pet trade: Game over*” organized by the Eurogroup for animals and the Croatian Presidency of the European Council (Bruxelles 21 April 2020) and it has been listed among the recommendations for the European Union as follows: “*Other enforcement projects should be funded under the European Regional Development Fund and Internal Security Fund based on the Bio-crime model at key borders crossed by the puppy trade using the links between animal and human health and the 43 IPCCCs*” (1).

**Figure 6:**
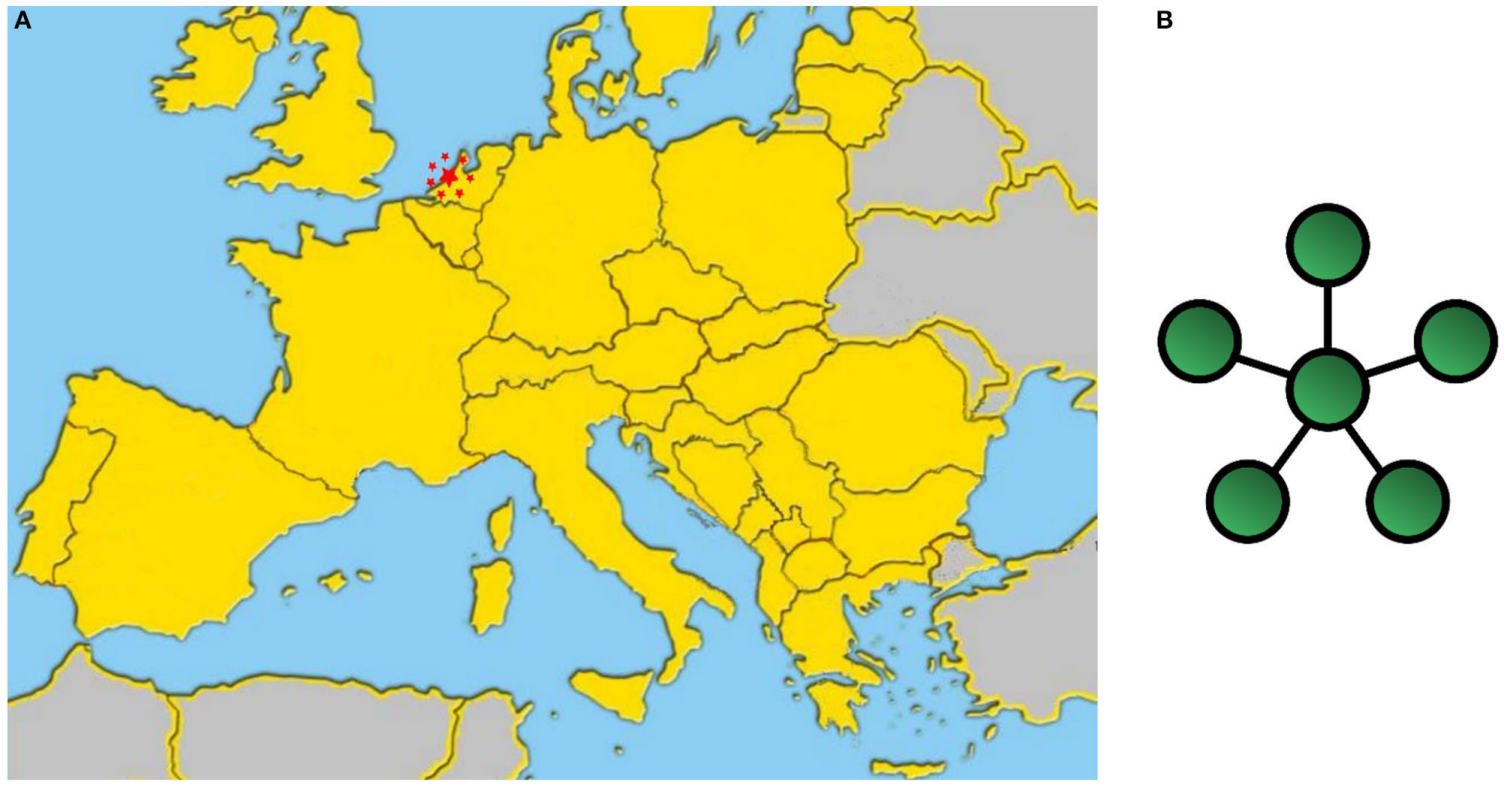
**(A)** The “centralized star network” approach uses a single EU task force that brings together all the stakeholders of the EU Member States; **(B)** Network topology of a star network (icon 6B from Wikimedia Commons, the free media repository).

There are at least two main approaches that could help to contrast the illegal animal trade and bio-terrorism. One “*centralized star network*” approach to solve the problem is to create a single EU task force, hosted by an existing European agency such as EUROPOL for instance, that brings together all the stakeholders of the EU Member States (see Figures 6A,B).

**Figure 7:**
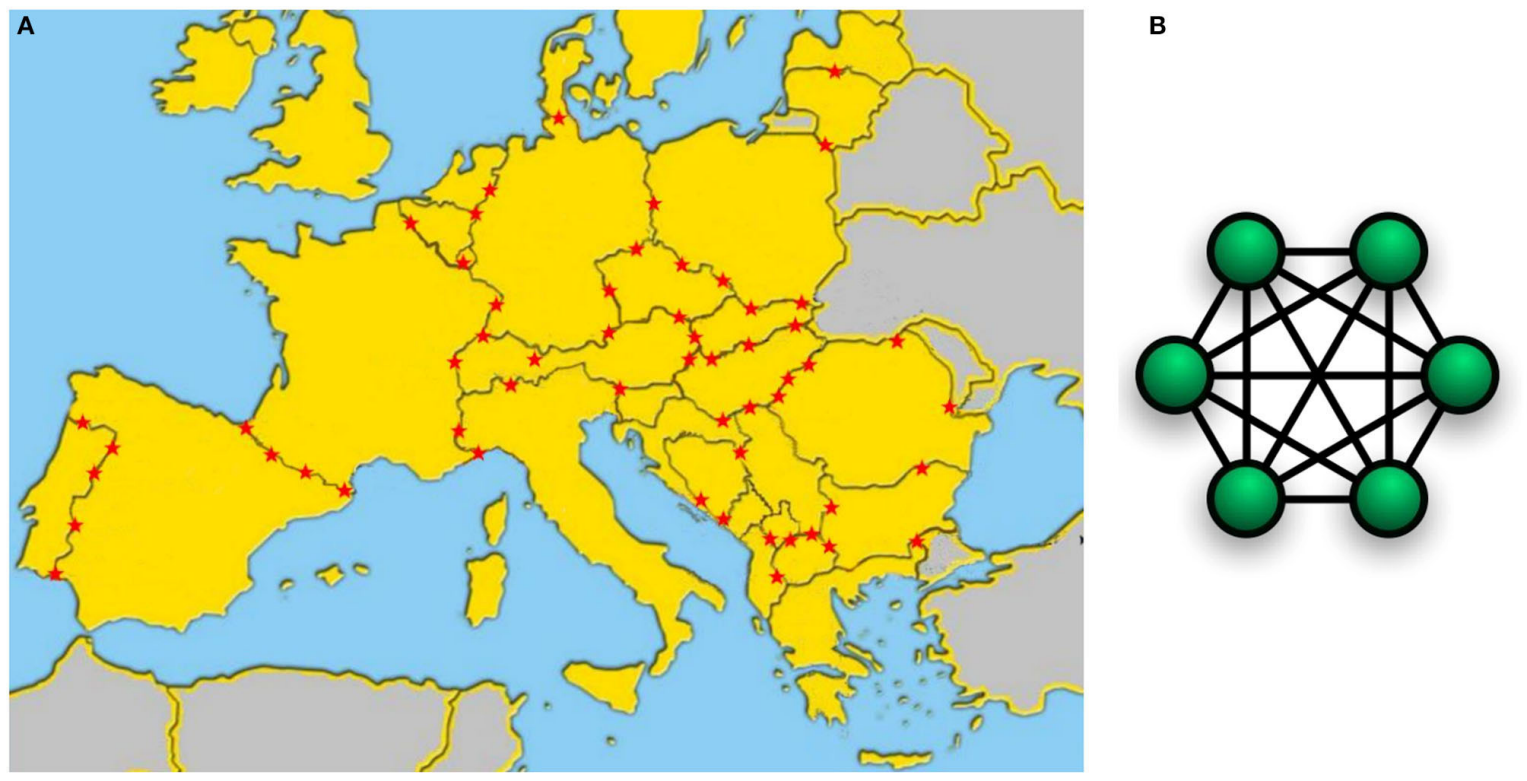
**(A)** The “Bio-crime model” can easily be scaled at European level by means of a “fully connected network” approach: the architecture of the Bio-crime model that connects Veterinary Public Health, Justice, Law Enforcement and Customs at a cross-border level as described in Figure 4, is replicated in every International Police and Custom Cooperation Centre (IPCCCs) of Europe (identified with a red star). Each IPCCC is already connected one to each other; **(B)** (right) Network topology of the “fully connected network” used by the Bio-crime model where all nodes (IPCCCs) are interconnected (icon 7B from Wikimedia Commons, the free media repository).

Another approach, represented by the “Bio-crime model” can easily be scaled at European level by means of a “*fully connected network*” perspective: the architecture of such model connecting Veterinary Public Health, Justice, Law Enforcements and Customs at a cross-border level as described in Figure 5, is replicated in every International Police and Custom Cooperation Centre (IPCCCs) of Europe that are already connected one to each other (see also Figures 7A,B).

Both approaches have advantages and disadvantages: a “*centralized star network*” provides a global view of what is happening all over Europe and allows the implementation of common strategies; however, at the same time its centralized architecture limits its connections with the fields. Furthermore, the fault/delay response of the central node (Central Agency) will stop the whole network activity. On the other hand, the “*fully connected network*” of the “Bio-crime model” is much more responsive and appropriate to investigate, and it guarantees more rapid decisions at local level. According to our experience, the “*centralized star network*” EU task force and the Bio-crime “*fully connected network*” models are not opposed but complementary because one node represented by a IPCCC of the Bio-crime model can acquire, even if temporarily, a higher hierarchical level and coordinate the activities of the others. In case of fault/slowdown of the lead node (Central Agency), the highest hierarchy level can be immediately shifted to an IPCCC, thus keeping the network always active and efficient.

### Illegal Pet Trade Phenomena at Cross Border Level

The illegal pet trade data collected by the Bio-crime program allows us to have an exhaustive picture of this criminal activity that transports pets from Eastern countries to Europe and sells them on black markets. Over time, the countries of origin of the illegal pet trade have changed their attitude and have become more involved, not only non-European countries relatively close to the EU borders such as Ukraine, but also countries such as Russia or even China. The number of events and the prevalence of the species are quite constant over time. Dog and cat puppies and birds are the pet species most subject to illegal pet trade. Most of the confiscated animals don't have an identification device, pet passport or valid rabies vaccination and they represent a serious health risk. In order to avoid the introduction of infectious diseases into the EU countries, 77.5% of the 652 puppies confiscated between December 2017 and July 2020 were placed in sanitary quarantine by the local Veterinary Officers. Thanks to the financial support from the Bio-crime project, it was possible to perform laboratory tests to all the confiscated animals and carry out necropsies on those that had died during transport or while in sanitary quarantine. It is difficult to briefly summarize the results of the laboratory survey performed on the confiscated animals although, it was surprising to discover that (i) almost 100% of the confiscated cat and dog puppies were positive for *Toxocara* sp., an intestinal zoonotic nematode transmissible to humans; (ii) about 5.5% of the dog and cat puppies were positive to *Salmonella* sp.; (iii) serological investigation displayed failure in post-rabies vaccination immunity in about 75% of total analyzed, a percentage even worse than previously described (22) while *Chlamydia psittaci*, Canine distemper and Parvovirus have been isolated quite frequently in the target species. Although most zoonoses evolve from wildlife hosts, most of them need an intermediate domestic/pet hosts transmission to be able to spread to human (23–25). For this reason, the choice of focusing the health monitoring procedures on confiscated pets coming from the illegal trade developed by the Bio-crime project can improve the effectiveness of human and animal health prevention procedures and reduce the risk of incursion of zoonotic and exotic animal diseases into the EU Member States (4, 26).

### Illegal Pet Trade, On-Line Illegal Pet Trade and Bio-Terrorism

The economic impact of a zoonotic event (natural, accidental, or deliberate) is difficult to assess because it changes according to the different pathogen. However, small-scale events can generate high economical losses (21, 27) and the example of Chlamydia-infected parrots mentioned in the results section confirms how true this statement is. The total value of the parrots and the truck used to transport these animals did not exceed €5,000 while the damage they could have caused to the health system if they had been sold on the market ranged between €35 and €420 M. A single event of this kind, regardless of whether the introduction of the pathogen is natural, accidental or deliberate, can lead to a default of the health system in a whole Region.

Through the simultaneous use of different tools, the Bio-crime program has contributed to reduce the risk of natural, accidental or deliberate introduction of pathogens dangerous for human and animal health. First, suspicious transports were examined with great attention posed to the species of animals transported, to the infectious isolated diseases and, in particular to the Cost-Benefit Ratio (CBR) between the value of the transported pets and the distance traveled by car. For instance, transporting only few puppies in a vehicle traveling for days from Asia to Europe is a suspicious behavior, as the sale value of the puppies on the market does not even cover the cost the fuel.

Furthermore, the online trade of dogs, cats and other pet species was monitored simultaneously with a veterinary medical intelligence approach. This trade, and digital technologies in general, can pose significant challenges for the competent authorities of the Member States operating within national boundaries, particularly as their systems of official controls may not be adequately adapted to deal with the rapidly evolving character and cross-boundary nature of internet commerce (28). Open Source Intelligence (OSINT) tools were also used to monitor the on-line illegal pet trade, while computational linguistic and sentiment analysis tools were used to profile illegal animal traders and to analyze bio-terroristic claims.

### The Cost-Benefit Ratio (CBR) of the Program Investments of the Bio-Crime Project

The results of a 3 years cross-border cooperation extended also to the health sector confirm what has already been widely reported in the scientific literature, namely that local public health interventions are highly cost-saving. Free trainings generate a saving rate higher than the entire project budget with a Cost-Benefit Ratio that is almost 20 times the budget used while the cost avoidance related to the spread of infectious diseases transmissible from animals to humans can generate a Cost-Benefit Ratio that for a single criminal event can reach up to 313 times the whole budget of the project. Therefore, cuts to public health budgets in high income countries therefore represent a false economy, and are likely to generate billions of Euro of additional costs to health services and the wider economy (29).

## Conclusions

The prevention and control of zoonotic diseases related to the illegal pet trade is not only a matter of the cooperation architecture model but requires a strong communication network and a continuous training education program for all the stakeholders involved. The public health infrastructure must look beyond passive surveillance of acute animal disease events to build capacity for active surveillance and intervention efforts to detect and control on-going outbreaks of disease in domestic and wild animal populations (30). In fact, there are no short cuts, because you can control disease only with laboratory capacity, workforce training (epidemiology, clinical, laboratory, IT, …), surveillance and network communications across political boundaries (31, 32). It will be more and more important to use a medical intelligence approach, including also social media content analysis, during the processes of data collection, data analysis and data sharing at cross-border level for a better prevention, monitoring and control of zoonotic diseases (21, 33).

“Although it is difficult to make predictions, especially those about the future” (quot. by Niels Bohr), the use of predictive models for risk assessment in veterinary medicine is becoming increasingly important as demonstrated for instance by the “spatial risk assessment model framework for incursion of exotic animal disease into the European Union Member States” by Simons et al. (34) and by the Handbook for the Assessment of Capacities at the Human-Animal Interface (35, 36). The Bio-crime model of cross-border cooperation is placed within the context of Organizational modeling that integrates organizational structure, goals, behavior and components and has been developed to improve the veterinary health prevention procedures at cross-border level. Such model proves that an increase in communication and data sharing on a horizontal level among public officers belonging to different disciplines like Justice, Law enforcements, Customs, Veterinary public health, improves the early-warning system, increasing the capacity to detect, report, assess and respond to the entry of zoonotic infectious diseases into the EU Member States according to the International Health Regulation standard (35, 36).

Despite that fact that 60% of existing human infectious diseases are zoonoses and most of the bioterrorism threat agents are zoonotic disease agents, veterinary medical expertise is scarcely present in biohazard training and education programs for law enforcement agencies and public officials. We hope that in the near future, collaboration and data sharing between Veterinary public health, Justice, Law Enforcements and Customs engaged in the fight against illegal animal trade and agro/bio-terrorism, will increase (21). In fact, “without data you're simply another person with an opinion” (quot. W.E. Deming).

## Data Availability Statement

The datasets generated for this study can be found in online repositories. The names of the repository/repositories and accession number(s) can be found at: https://www.biocrime.org/frontiers-data-repository.

## Ethics Statement

Ethical review and approval was not required for the animal study because scientific data related to the pet animals mentioned in the article come from clinical and laboratory examinations performed by the Public Veterinary Health Authorities on confiscated pets. The only aim of these veterinary procedures was to diagnose diseases and treating ill confiscated pets as required by EU and national MS legislation for the protection and welfare of animals/zoonoses protection. Confiscated pets have not been subjected to any further procedures other than those required by law. According to current EU and MS legislation, routine public health veterinary procedures on confiscated pets from the illegal animal trade don't fall under the legislation on laboratory animals research and don't require the authorization of an ethical committee.

## Author Contributions

PZu, M-CR, MK, WG, GM, HM, PW, PDF, EC, and JH conceived of the idea of developing a cross-border model of cooperation. PZu wrote the manuscript proof. All authors discussed the results and contributed to the final version of the manuscript.

## Conflict of Interest

The authors declare that the research was conducted in the absence of any commercial or financial relationships that could be construed as a potential conflict of interest.
